# Predictors of Treatment Outcomes among Pediatric Patients Hospitalized with Pneumonia in Tikur Anbessa Specialized Hospital, Addis Ababa, Ethiopia

**DOI:** 10.1155/2021/6690622

**Published:** 2021-04-20

**Authors:** Hilina Tsegaw, Mohammed Yimam, Dejen Nureye, Workineh Woldeselassie, Solomon Hambisa

**Affiliations:** ^1^School of Pharmacy, College of Medicine and Health Sciences, Mizan-Tepi University, Mizan-Teferi, Ethiopia; ^2^Department of Pharmacy, College of Health Sciences, Debre Brehan University, Debre Brehan, Ethiopia; ^3^Department of Pharmacy, College of Medicine and Health Sciences, Ambo University, Ambo, Ethiopia

## Abstract

**Background:**

Pneumonia remains the leading cause of hospitalization and mortality in young children in low- and middle-income countries. This study is aimed to assess predictors of treatment outcomes among pediatric patients hospitalized with pneumonia in Tikur Anbessa Specialized Hospital, Addis Ababa, Ethiopia.

**Methods:**

A facility-based retrospective cross-sectional study was conducted among pediatric patients admitted with pneumonia, considering patient medical charts recorded for a 1-year period from February 2017 to February 2018. The sample size was computed based on a single population proportion formula and giving a total sample size of 207. The systematic random sampling method was employed to select patient cards from the sampling frame. The data extraction format was used to extract any relevant information from patient chart. The processed data were analyzed by using Statistical Package for Social Sciences (SPSS, version 21). Descriptive statistics were used to summarize the patients' baseline characteristics and related information. The logistic regression model was fitted to determine factors associated with treatment outcomes. To identify predictors of poor treatment outcome, the level of significance was set at *P* < 0.05.

**Results:**

From a total of 207 patient charts reviewed, more than half (55.6%) of the study participants were males. Majority of patients, 130 (62.8%), were in the age range of 1 month–11 months. Furthermore, 191 (92.3%) patients had good treatment outcome. Patients who treated with ceftriaxone + azithromycin were less likely to have poor treatment outcome compared with patients who were placed on crystalline penicillin (AOR = 0.86, 95% CI 0.01–0.83). On the contrary, patients who stayed ≥ 8 days were about 14.3 times more likely to have poor treatment outcome compared with patients who stayed ≤ 3 days (AOR = 14.3, 95% CI 1.35–151.1).

**Conclusion:**

Even though the study revealed good treatment outcome among the pediatric patients, particular consideration should be given to children in need of other interventions.

## 1. Background

Pneumonia is a severe form of acute respiratory infection known to affect the lungs [1, 2]. It remains the leading cause of hospitalization and mortality in young children in low- and middle-income countries as per the 2016 UNICEF annual report and is estimated to cause 896,000 deaths of the total 5.6 million deaths [3, 4]. In Ethiopia, pneumonia is a substantial cause of morbidity and mortality in the pediatric population, and regardless of improved preventive strategies, approximately 4 million childhood pneumonia cases are occurring annually [5].

A number of infectious agents are responsible for childhood pneumonia, including viruses, bacteria, and fungi [1, 2]. The respiratory syncytial virus, *Streptococcus pneumoniae*, and *Haemophilus influenzae* are the leading causes of childhood pneumonia, with the latter two being preventable through administration of vaccines [6]. The main risk factors for pediatric pneumonia include poor socioeconomic status, not being able to breastfed, malnutrition, indoor air pollution, household crowding, low birth weight, incomplete immunization schemes, HIV, prolonged duration of illness, and the presence of underlying chronic illness such as underlying heart disease [7–9].

Although under-five mortality was consistently reduced over the past 20 years, few progresses in reducing neonatal mortality have been done [10]. Pediatric patients who receive effective and timely treatment recover without complication. However, patients with no access to effective intervention are at high risk of developing complications and death. The major complications of pneumonia include lung abscess, pleural effusion and empyema, and pneumothorax [11].

Most guidelines recommend the use of a combination of *β*-lactam antibiotics with a macrolide or monotherapy with respiratory fluoroquinolone, but not *β*-lactam monotherapy in treatment of pneumonia cases. This is especially for patients who are admitted to medical ward of the health institutions [12]. Likewise, treatment regimens are needed to be chosen based on their efficacy in local settings; for instance, some areas may have a high burden of resistance to certain antibiotics, rendering these antibiotics are less effective for management of pneumonia. It is also recommendable to consider high-risk groups, such as malnourished or HIV-positive children to adapt their treatment approaches according to their medical cases for the better outcome [13, 14].

Despite the availability of effective management and treatment tools for pneumonia [15], poor response to initial empirical treatment of community-acquired pneumonia represents a major challenge for clinicians and requires early identification and intervention. The incidence of treatment failure in community-acquired pneumonia (CAP) is 10 to 15%, and the mortality is increased nearly fivefold. Resistant and unusual microorganisms and noninfectious causes are responsible for the treatment failure. Risk factors for the poor treatment outcomes are related to the initial severity of the disease, the presence of comorbidity, the microorganism involved, and the antimicrobial treatment administered during pharmacotherapy [16].

Several studies have reported variation in extent of treatment outcome among pediatric populations admitted with pneumonia [5, 17, 18]. Furthermore, the investigation of treatment outcome among patients with pneumonia would have paramount importance to demonstrate the extent of pneumonia management and care in health care settings [5]. Identifying factors associated with the treatment outcomes among pediatric patients hospitalized with pneumonia also play a crucial role for proper planning and intervention, particularly in developing countries like Ethiopia where prevalence of infectious diseases is high. Consequently, this study is aimed to assess predictors of treatment outcomes among pediatric patients hospitalized with pneumonia in Tikur Anbessa Specialized Hospital (TASH), Addis Ababa, Ethiopia.

## 2. Methods and Materials

### 2.1. Study Setting and Study Design

A facility-based retrospective cross-sectional study was conducted among pediatric patients hospitalized with pneumonia at TASH, Addis Ababa, Ethiopia. TASH is found in the capital city of Ethiopia, Addis Ababa, and it is among the oldest and pioneering teaching referral hospitals with a capacity of 700 beds. TASH has a range of specialties including pediatrics, surgery, gynecology, and psychiatry. The pediatric unit provides emergency service for patients who are referred from different health institutions, and it is the main portal of entry for children who need prompt admission and management.

### 2.2. Sample Size and Sampling Procedure

For this particular study, patient medical charts recorded for a 1-year period from February 2017 to February 2018 were reviewed, and the sample size was computed based on the single population proportion formula, taking the assumption of 5% margin of error, 95% confidence interval, and 16% prevalence of death from pediatric pneumonia [3] and giving a total sample size of 207. The systematic random sampling method was employed to select patient cards, and the sampling interval was calculated by dividing patient charts kept for one year by actual sample size.

Patients with age ≤14 years admitted with clinical diagnosis of pneumonia in a pediatric ward of TASH were included in our study. Pediatric patients with uncertainty in the diagnosis of pneumonia, patients with incomplete medical records, patients who died before they start treatment, and patients referred to other health care facilities were excluded from the study.

### 2.3. Data Collection Tool and Procedure

The data extraction format was developed to extract any relevant information from patient chart regarding patient sociodemographic characteristics, clinical characteristics of the patients including diagnostic method(s) used for identification of the disease, pertinent laboratory findings, comorbidities, medications administered, type and severity of the disease, patient's discharge information (treatment outcome), and duration of hospital stay. Data collection was performed by well-trained three pharmacy professionals, and completeness of collected data was checked by the supervisors.

#### 2.3.1. Data Analysis

The processed data were analyzed by using Statistical Package for Social Sciences (SPSS, version 21). Descriptive statistics such as frequency, percentage, and mean were used to summarize the patients' baseline characteristics and related information. The logistic regression model was fitted to determine factors associated with treatment outcomes. Variables with *P* value ≤0.25 on bivariate logistic regression were entered into multivariate logistic regression, and to identify predictors of poor treatment outcome, the level of significance was set at *P* < 0.05.

#### 2.3.2. Ethical Consideration

Ethical clearance was obtained from the Ethical review board of Mizan-Tepi University, College of Medicine and Health Sciences. Official permission to access patients' data was granted from the hospital management. Participants' information was kept confidential and anonymous. Patient medical charts were handled in an appropriate manner.

### 2.4. Operational Definitions

#### 2.4.1. Treatment Outcome

Result was obtained after the use of a drug. It may be cure, death, and complication.

#### 2.4.2. Good Treatment Outcome

It represents the discharge from hospital on the grounds of clinical improvement.

#### 2.4.3. Poor Treatment Outcome

It is defined as the occurrence of death and complications or an intensive care unit (ICU) admission.

## 3. Results

### 3.1. Demographic Characteristics

From a total of 207 patient charts reviewed, more than half (55.6%) of the patients were males. The majority of patients, 130 (62.8%), were in the age range of 1 month to 11 months. Two-thirds (66.7%) of the participants were weighed in the range of 6 to 10 kg (Table 1).

### 3.2. Clinical Characteristics of the Study Participants

Regarding the types of pneumonia, most patients (88.9%) had been diagnosed with community-acquired pneumonia (CAP), followed by hospital-acquired pneumonia (HAP) (5.3%). The severity of pneumonia was determined as severe pneumonia in most of the participants (88.9%), while 21 (10%) had been depicted as nonsevere one. From all pediatrics admitted with pneumonia, one hundred forty-four (69.6%) were diagnosed merely based on complete blood count. However, around one-fourth (24.6%) of the cases were confirmed together with chest radiography. Causative microorganisms were not identified in most patients (Table 2).

As presented in Table 2, the study revealed that the majority (56.5%) of patients had comorbid diseases besides pneumonia. Congestive heart failure (CHF) and hyperactive airway disease (HAAD) were the most common comorbidities in the study population, which accounts for 41 (19.8%) and 20 (9.7%), respectively. Eighty-one (39%) patients stayed in hospital for 4–5 days, while merely around one-fifth (17.4%) of study participants were admitted in hospital beyond one week. The mean duration of the hospital stay was 2.52 days.

### 3.3. Medication-Related Characteristics

Patients were treated with a variety of parenteral antibiotics during their hospital stay. The most frequently used antibiotics were parenteral crystalline penicillin (111 (53.6%)), followed by ceftriaxone (46 (22.2%)). Paracetamol was the most commonly prescribed comedication (27 (11%)). From the total number of children treated for pneumonia, most (85%) of the participants were discharged with oral medications. Out of the total prescribed medications, amoxicillin-clavulanate (Augmentin) (57 (27.5%)) and amoxicillin (56 (27.1%)) were the most commonly prescribed oral medications (Table 3).

### 3.4. Factors Associated with Treatment Outcome

Out of 207 pediatric patients, the majority (191 (92.3%)) of them were found to have a good treatment outcome. In contrary, 16 (7.7%) of the participants had a poor treatment outcome (Table 2). The results of univariate logistic regression analysis depicted that patients age group between 1 year and 3 years (COR = 0.022, 95% CI: 0.001–0.67), patients whose weight was between 6 and 10 kg (COR = 0.22, 95% CI: 0.05–0.67), patients who were treated with vancomycin (COR = 20.1, 95% CI: 3.32–121.3), patients who were treated with ceftriaxone + azithromycin (COR = 7.6, 95% CI: 1.8–49.2), patients who were treated with ceftriaxone + metronidazole (COR = 10.7, 95% CI: 1.6–72.9), and patients who stayed in hospital beyond one week (COR = 3.57, 95% CI: 1.9–18.4) were significantly associated with poor treatment outcome.

Among the variables entered to multivariable logistic regression, treating patients with the ceftriaxone plus azithromycin and duration of hospital stay for ≥ 8 days were independent predictors of poor treatment outcome. Accordingly, patients who were treated with ceftriaxone + azithromycin were less likely to have a poor treatment outcome compared with patients who were placed on crystalline penicillin (AOR = 0.86, 95% CI: 0.01–0.83). On the contrary, patients who stayed for ≥ 8 days in hospital were 14.3 times more likely to have a poor treatment outcome compared with patients who stayed ≤ 3 days in hospital (AOR = 14.3, 95% CI: 1.35–151.1) (Table 4).

## 4. Discussion

This study revealed a high proportion of good treatment outcome among pediatric patients diagnosed with pneumonia. This finding is comparable to the multicenter study done in the United States of America, in which 97.6% of patients showed clinically significant improvements [17]. However, the finding is higher than the result that came out from Rabat, Morocco, where 72.8% of children had a poor prognosis [18]. Similarly, the finding of the present study is higher than the result from the Nekemte Referral Hospital, Ethiopia, in which poor treatment was observed in 69.4% of pediatric patients [5]. This discrepancy might have been related to the variation in the level of care given by the hospitals for the patients.

On the contrary, poor treatment outcome reported in some pediatric patients could be due to the fact that most patients visiting TASH are referred from other neighboring hospitals and terminally ill. Likewise, the presence of a high proportion of comorbidities like HIV, TB, heart failure, and anemia may result to poor prognosis. It is also interesting to note that both good treatment outcome and poor treatment outcome revealed in the study were within the minimum standard set of the WHO guideline for management of severe pneumonia [19].

For a large number of pediatric patients diagnosed with pneumonia, crystalline penicillin was prescribed followed by ceftriaxone injections. In connection to this, the result is higher than another study obtained from Ethiopia, in which crystalline penicillin was ordered for 36% of patients [5]. The variation might be attributed to the differences in the study settings. In TASH, patients are most of the time given a care by the specialists and those health care providers were trained at an advanced level. Furthermore, the higher utilization of crystalline penicillin and ceftriaxone attributed to the fact that these drugs have been recognized as a drug of choice owing to its excellent bioavailability, effectiveness, and low toxicity profile [18, 20, 21]. Moreover, a recent randomized controlled trial and a preceding systematic review revealed that *β*-lactam antibiotics alone may be as effective as respiratory fluoroquinolones or combination regimens in the treatment of hospitalized patients diagnosed with CAP [22, 23]. Conversely, this study demonstrated that only a small fraction of patients was treated with ceftriaxone plus azithromycin, which is an alternative treatment for the pneumonia caused by resistant pathogen as stipulated in the Ethiopian national treatment guideline [20].

In the present study, treating pediatric patients with ceftriaxone and azithromycin was significantly associated with poor treatment outcome in the univariate and multivariate logistic regression models. It was observed that patients who treated with ceftriaxone + azithromycin were less likely to have a poor treatment outcome compared with patients who were placed on crystalline penicillin. Several studies have revealed that, in severely ill community-acquired pneumonia patients, adding a macrolide to the antibiotic regimen is associated with better clinical outcomes [24, 25]. Furthermore, macrolide antibiotics should be used if either mycoplasma or chlamydia pneumonia is suspected or in very severe disease [26]. Similarly, ceftriaxone is recommended for beta-lactamase-producing *Haemophilus influenzae* and rapidly progressing multilobar disease or pneumatoceles [27].

It was highlighted that a mean duration of hospitalization in our study was shorter. This study finding is lower as compared to similar finding that came out from New Delhi, India, where the mean length of hospital stay was 6.8 days [28]. Prolonged hospitalization was a significant predictor of poor treatment outcome. It was observed that patients hospitalized for ≥ 8 days were about 14.3 times more likely to have a poor treatment outcome compared with patients admitted for  ≤ 3 days. This could be due to the fact that longer duration of hospital stay could be related to overcrowding, higher risk of developing hospital-acquired infection, and complications of bacterial pneumonia like persistent effusions, empyema, pulmonary abscess, and respiratory distress sepsis. Besides, the finding is slightly concurrent with the study done in Rabat, Morocco, that reported 9.96 days of hospital stay were significantly (*P*=0.001) associated with poor treatment outcomes [18].

## 5. Limitation of the Study

The study has several limitations that should be taken into account while interpreting the results. As the data were collected retrospectively, a result does not show a strong causal effect between the variables of interest. The study was conducted at only a single facility, so caution should be taken in extrapolating the results of the study. A larger-scale and multicenter survey that includes more diverse participants is needed to provide more accurate finding.

## 6. Conclusion

The present study revealed that the majority of the pediatric patients admitted with pneumonia had a good treatment outcome. Treatment with ceftriaxone and azithromycin and prolonged hospitalization were a significant predictor of treatment outcome. Patients treated with ceftriaxone plus azithromycin showed a better treatment outcome than patients treated with crystalline penicillin alone. Longer duration of hospital stay was more likely associated with poor prognosis. Therefore, particular consideration should be given to children in need of other interventions.

## Figures and Tables

**Table 1:** Demographic characteristics of the children admitted with pneumonia at pediatric ward of Tikur Anbessa Specialized Hospital from February 2017 to February 2018, Addis Ababa, Ethiopia (*N* = 207).

Demographic variable	Frequency (%)
*Sex*	
Male	115 (55.6%)
Female	92 (44.4%)

*Age*	
<1 month	2 (1%)
1 month–11 months	130 (62.8%)
1 year–<3 years	46 (22.2%)
3 years–<6 years	16 (7.7%)
>6 years	13 (6.3%)

*Weight (kg)*	
1–5 kg	23 (11.1%)
6–10 kg	138 (66.7%)
11–15 kg	30 (14.5%)
>16 kg	16 (7.7%)

**Table 2:** Clinical characteristics of the study participants admitted with pneumonia in pediatric ward of Tikur Anbessa Specialized Hospital from February 2017 to February 2018, Addis Ababa, Ethiopia.

Clinical variable	Frequency (%)
*Comorbid disease*	
No	90 (43.5%)
Yes	117 (56.5%)

*Specific comorbidities*	
HAAD	41 (19.8%)
Anemia	11 (5.3%)
Malnutrition	14 (6.8%)
CHF	20 (9.7%)
AGE	18 (8.7%)
Others^*∗*^	13 (6.3%)

*Severity class of pneumonia*	
Very severe	2 (1%)
Severe	184 (88.9%)
Nonsevere	21 (10%)

*Types of pneumonia*	
CAP	184 (88.9%)
HAP	11 (5.3%)
Aspiration pneumonia	6 (2.9%)
Recurrent pneumonia	6 (2.9%)

*Diagnostic method*	
Complete blood count	144 (69.6%)
Complete blood count and chest radiography	51 (24.6%)
Culture	10 (4.8%)
Chest radiography	2 (1%)

*Hospital duration (days)*	
≤3	27 (13%)
4–5	81 (39%)
6–7	63 (30.4%)
≥8	36 (17.4%)

*Treatment outcome*	
Discharged with improvement	191 (92.3%)
Occurrence of death and complications	11 (5.3%)
Intensive care unit admission	5 (2.4%)

^*∗*^HIV/AIDS, hypertension, pyogenic meningitis, and disseminated TB.

**Table 3:** Patterns of medication utilization among children treated for pneumonia at the pediatric ward of Tikur Anbessa Specialized Hospital from February 2017 to February 2018, Addis Ababa, Ethiopia.

Patterns of medication utilization	Frequency (%)
*Types of antibiotic(s) administered*	
Crystalline penicillin	111 (53.6%)
Ampicillin + gentamycin	12 (5.8%)
Ceftriaxone	46 (22.2%)
Ceftriaxone + gentamycin	15 (7.2%)
Ceftriaxone + azithromycin	9 (4.3%)
Vancomycin	7 (3.4%)
Ceftriaxone + metronidazole	7 (3.4%)

*Comedication*	
No	71 (34.3%)
Yes	136 (65.7%)

Types of comedication	
Paracetamol	53 (25.6%)
Salbutamol puff	19 (9.2%)
Hydrocortisone and salbutamol	24 (11.6%)
Furosemide and spironolactone	10 (4.8%)
Others^*∗*^	30 (14.8%)

*Patient discharge medication*	
No	31 (15%)
Yes	176 (85%)

*Types of discharge medication*	
Amoxicillin-clavulanate	57 (27.5%)
Amoxicillin syrup	56 (27.1%)
Amoxicillin + paracetamol	14 (6.8%)
Amoxicillin + salbutamol puff	14 (6.8%)
Amoxicillin-clavulanate + salbutamol puff	7 (3.4%)
Amoxicillin-clavulanate + paracetamol	10 (4.8%)
Others	18 (8.7%)

^*∗*^Haem up syrup, rifampin, isoniazid, pyrazinamide, and ethambutol.

**Table 4:** Predictors of treatment outcomes among children treated for pneumonia at the pediatric ward of Tikur Anbessa Specialized Hospital from February 2017 to February 2018, Addis Ababa, Ethiopia.

Variable		Treatment outcome		Corollary (95% CI)	AOR (95% CI)
		Good	Poor		
Age	<1 month	1	1	1	1
	1 month–1 year	121	9	0.074 (0.004–1.3)	4.4 (0.015–13.2)
	1 year–3 years	45	1	0.022 (0.001–.67)^*∗*^	0.77 (0.01–73.2)
	3 years–6 years	14	2	0.143 (0.006–3.3)	0.27 (0.003–26.8)
	>6 years	10	2	0.300 (0.014–6.4)	1.2 (0.05–27.7)

Weight (kg)	1–5 kg	19	4	1	1
	6–10 kg	132	6	0.216 (0.056–0.84)^*∗*^	1.139 (0.11–118.3)
	11–15 kg	28	2	0.339 (0.056–2.041)	0.2.43 (0.03–17.6)
	>16 kg	12	4	1.58 (0.332–7.56)	0.396 (0.013–12.4)

Antibiotic administered	Crystalline penicillin	107	4	1	1
	Ampicillin + gentamycin	11	1	2.43 (0.25–23.7)	1.83 (0.072–46.57)
	Ceftriaxone	43	3	1.86 (0.40–8.6)	1.61 (0.24–10.96)
	Ceftriaxone + gentamycin	14	1	1.91 (0.19–18.3)	0.887 (0.08–9.4)
	Ceftriaxone + azithromycin	7	2	7.6 (1.8–49.2)^*∗*^	0.086 (0.01–0.83)^*∗*^
	Vancomycin	4	3	20.1 (3.32–121.3)^*∗*^	0.123 (0.011–1.35)
	Ceftriaxone + metronidazole	5	2	10.7 (1.6–72.9)^*∗*^	0.241 (0.024–2.4)

Hospital stay (days)	≤3	25	2	1	1
	4–5	77	4	0.65 (0.112–3.76)	0.828 (0.137–5.01
	6–7	61	2	0.41 (0.055–31)^*∗*^	3.73 (0.59–23.4
	≥8	28	8	3.57 (1.9–18.4)	14.30 (1.35–51.1)^*∗*^

## Data Availability

The authors declare that the supporting data on which conclusions of this manuscript are fully described within the manuscript.

## Abbreviations

AOR: Adjusted odds ratio
AGE: Acute gastroenteritis
CAP: Community-acquired pneumonia
CI: Confidence interval
COR: Crude odds ratio
CHF: Congestive heart failure
HAP: Hospital-acquired pneumonia
HAAD: Hyperactive airway disease
ICU: Intensive care unit
TASH: Tikur Anbessa Specialized Hospital
TB: Tuberculosis.
